# Aptamers: A Promosing Tool for Ochratoxin A Detection in Food Analysis

**DOI:** 10.3390/toxins5111988

**Published:** 2013-11-05

**Authors:** Amina Rhouati, Cheng Yang, Akhtar Hayat, Jean-Louis Marty

**Affiliations:** 1 IMAGES, Université de Perpignan, 52 Avenue Paul Alduy, Perpignan Cedex 66860, France; E-Mails: amina.rhouati@gmail.com (A.R.); yangcheng.chem@gmail.com (C.Y.); 2 Department of Chemistry and Biomolecular science, Clarkson University, Potsdam, NY 13699, USA; E-Mail: ahayat@clarkson.edu

**Keywords:** ochratoxin A, aptamer, purification, detection, analysis

## Abstract

The contamination of food and feed by mycotoxins has become an increasingly serious problem. Mycotoxins represent a major risk to human and animal health, as well as economics. Herein, we focus on Ochratoxin A (OTA), which is one of the most common mycotoxins contaminating feed and foodstuffs. OTA is a secondary metabolite produced by various *Aspergillus* and *Penicillium* strains. Upon ingestion, OTA has a number of acute and chronic toxic effects. It is nephrotoxic, teratogenic, immunosuppressive, and carcinogenic (group 2B). As a consequence, some regulatory limits have been introduced on the levels of OTA in several commodities. The toxic nature of OTA demands highly sensitive and selective monitoring techniques to protect human and animal health. As alternative to traditional analytical techniques, biochemical methods for OTA analysis have attained great interest in the last few decades. They are mainly based on the integration of antibodies or aptamers as biorecognition elements in sensing platforms. However, aptamers have gained more attention in affinity-based assays because of their high affinity, specificity, stability, and their easy chemical synthesis. In this brief review, we present an overview of aptamer-based assays and their applications in OTA purification and detection, appeared in the literature in the last five years.

## 1. Introduction

Nowadays, researchers focus increasingly on common food contaminants like mycotoxins. These ubiquitous toxins are produced by the secondary metabolism of fungi, mainly saprophytic molds. More than 400 mycotoxins, with different chemical structures and toxic effects, have been identified [1]. Among them, ochratoxin A (OTA) has attracted much attention because of its abundance and toxicity. OTA is produced by two main genera of filamentous fungi, *Aspergillus* and *Penicillium*, which grow on a variety of food products [2]. Structurally, OTA consists of a chlorinated dihydroisocoumarin moiety linked with a 7-carboxyl group to l-β-phenylalanine [3]. Therefore, it has an inhibitory effect on a number of enzymes that use phenylalanine as substrate. It is a mitochondrial poison, causing mitochondrial damage, oxidative burst, lipid peroxidation, and interferes with oxidative phosphorylation [4,5]. Moreover, reducing OTA levels from foodstuffs is not feasible because it is very resistant to food preparation procedures such as cooking, roasting, and fermenting [6]. 

The presence of OTA in food samples at very low concentration may induce toxic effects, therefore selective and sensitive detection of OTA is highly required in order to guarantee food safety and to minimize the potential risk to human and environmental health. Due to its fluorescent nature, OTA is generally determined by chromatographic techniques [7]. Despite their accuracy, these analytical methods are expansive, time consuming, and require qualified personnel. Alternatively, biochemical methods have received considerable attention owing to their low cost, simplicity, rapidity, high sensitivity, and possibility of miniaturization allowing real time detection. These methods are mainly based on the interaction between a recognition element and its target inducing a mechanism of molecular recognition. This interaction is then translated into a measurable signal by using a suitable transducer [8]. Antibodies have been widely used as affinity-based recognition elements and numerous immunochemical methods have been described for OTA detection in several food matrices [9,10,11,12]. However, being produced by animal immunization, antibodies are very expansive and unstable molecules. To overcome the various limitations of antibodies, an alternative approach may be adopted by the use of synthetic bioreceptors, such as aptamers and Molecularly Imprinted Polymers (MIPs). MIPs result from the polymerization of monomeric units in the presence of a template molecule. Aptamers are artificial nucleic acids produced by chemical synthesis. These promising recognition elements are characterized by high affinity and specificity to their targets. Indeed, in comparison to antibodies, aptamers, and MIPs are very stable and their production is easier and cost-effective. The first application of MIPs in OTA analysis has been reported in 2004 [13], while the first OTA-binding aptamer was reported in 2008 and, since then, this has been used in a large number of detection strategies [14].

The principal objective of this review is to discuss the different aptamer-based methods developed for OTA determination in food matrices. The use of aptamers in OTA detection and purification will be discussed as well as the advantages of aptamers over antibodies in such biochemical methods. 

## 2. Ochratoxin A

### 2.1. Occurrence and Effects

Ochratoxinogenesis can occur during crop growth, harvest, storage, or processing, which may lead to OTA contamination in several food and feedstuffs. This mycotoxin is widely found in cereals such as corn, wheat, barley, oats, and millet and cereal-derived products such as flour, bread, beer, and vodka [15,16,17,18]. After cereals, wine is considered as the second source of human consumption (10% to 15% of daily OTA intake) [19,20]. Recently, the contamination of wines and grape juices by OTA has been shown in different studies [21]. OTA has also been identified as a contaminant of coffee beans [22], cocoa [23], dried fruits [24], and spices [25] throughout the world. Moreover, OTA is a natural contaminant of farm animal feeds and it has been traced in meat, milk, and dairy products [26].

As a widespread food contaminant, OTA was found in human blood [26,27], milk [28], and urine [29,30,31]. This mycotoxin has several effects on animal and human health. The main harmful effect of OTA is its nephrotoxicity, as demonstrated by many studies on animals [30,31,32,33]. Additionally, OTA causes acute and chronic nephropathies to humans, and it is suspected to be the main etiological agent responsible for Balkan Endemic Nephropathy (NEB), a chronic tubulointerstitial renal disease [29,34,35]. Based on the carcinogenic potency of OTA to rodents, the international Agency of Research on Cancer (IARC) has considered it as a potential carcinogen for humans (group 2B) [4,36]. Immunotoxic and myelotoxic effects of OTA have been also reported. The immunosuppressant activity of OTA is characterized by alteration in number and activity of immune cells and size reduction of immune organs [37]. In addition, OTA induces diverse effects on hematopoietic progenitor proliferation [38]. Finally, OTA has been observed to be teratogenic with diverse fetus malformations in a number of animal models [39,40,41,42,43]. 

### 2.2. European Regulations

Based on the toxicological evaluation performed by scientific committee on food, the European Union has established a tolerable weekly intake of 120 ng of OTA per kg of body weight (Commission Regulation No. 594/2012) [44]. In addition, some directives have been introduced by the European Union in order to set the maximum permitted levels of OTA in foodstuffs such as cereals (5 µg/kg) and all cereal-derived products (3 µg/kg), roasted coffee and coffee products (5 µg/kg), grape juice and all types of wine (2 µg/L), dried fruits (10 µg/kg), and spices (15 µg/kg) (Commission Regulation No. 123/2005). Furthermore, the maximum tolerated amounts of OTA have been fixed in cereals used in the composition of feedstuffs (0.25 mg/kg), the complementary and complete feed for pork (0.05 mg/kg), and poultry (0.1 mg/kg) (Commission Regulation No. 576/2006) [45].

Moreover, in a Canadian study performed on various sex/age groups, a virtually safety dose of 4 ng/Kg bw per day was calculated [46].

## 3. Aptamers: Bioreceptors

The word aptamer is derived from the latin “aptus” “to fit” meaning a polymer that “fit” to its target [47]. Aptamers are artificial short single stranded oligonucleotides, either DNA or RNA, selected from a combinatorial library of sequences according to their ability to recognize a target with high affinity and specificity. They can be generated against various targets such as, proteins, drugs, organic, or inorganic molecules. Aptamers fold into well defined three dimensional structures and bind to their ligands by complementary shape interactions, they can incorporate small molecules into their nucleic acid structure or integrate into the structure of larger molecules [48]. Aptamers are suitable for applications based on molecular recognition as analytical, diagnostic, and therapeutic tools [49].

### 3.1. *In Vitro* Selection of Aptamers

Aptamers are generated through an *in vitro* selection procedure called SELEX (Systematic Evolution of Ligands by Exponential Enrichment). It consists of an iterative process (typically, 7 to 15 rounds) alternating between selection (related to the shape) and amplification (related to the sequence) of nucleic acid variants. First, an oligonucleotide combinatorial library is synthesized; each oligonucleotide contains a random central region of 20 to 80 nucleotides, flanked by a primer-binding region at each end [50]. During the selection, target molecules are incubated with the random library in an appropriate buffer and under certain conditions of pH and temperature for a given period of time. Then, free oligonucleotides are separated and bound-oligonucleotides are eluted. After selecting the oligonucleotides with the best affinity for the target, these sequences are amplified by PCR (Polymerase Chain Reaction) using primers corresponding to fixed regions of the library. In addition to the purpose of enrichment of the selected aptamer pool, it is also possible to attach modifications via special primers during the amplification step. After that, the enriched pool is available as double stranded DNA and single strand separation has to be carried out to start the next round of SELEX. By iteratively executing the procedures of selection and amplification, the complexity of the library is reduced and the strongest affinity binders are enriched. Finally, the individual binding molecules are determined by cloning the final pool into a bacterial vector and sequencing the individual colonies. However, it is difficult to identify optimal sequences from this pool using traditional cloning and sequencing approaches. Recently, a few studies have shown that the use of high-throughput sequencing in the screening of aptamers yields a powerful tool for the identification of aptamers [51]. Sequence alignments, secondary structure analysis and binding studies are required to identify the final sequence and the characteristics of the identified aptamer [52]. 

### 3.2. DNA Aptamers against Ochratoxin A

Two research groups have applied SELEX process for the screening of DNA aptamers against OTA. In 2008, Cruz-Agado and Penner have isolated the first aptamer of OTA, this aptamer designated 1.12.2 contains 36 nucleotides [14]. In the procedure followed by the authors, each selection cycle consisted of loading a library containing 10^15^ random oligonucleotide sequences onto an affinity column containing immobilized OTA. The column was then washed with binding buffer (BB), and an enriched fraction with putative binding ability to OTA was eluted through the addition of free OTA. This enriched library was amplified, the sense strands were recovered through the use of a biotinylated antisense primer, and the library was reapplied to fresh immobilized OTA columns. The aptamer was selected in a selection buffer (10 mM HEPES, pH 7.1, 20 mM NaCl, 5 mM KCl, 5 mM, MgCl_2_), where the dissociation constant was 0.2 µM. The authors have demonstrated that the aptamer does not bind molecules with structures similar to OTA such as *N*-acetylphenylalanine or warfarin and bound with a 100-fold less affinity to ochratoxin B. No secondary structure has been suggested for this aptamer, but doublets or triplets of guanine present at more than four times in the sequence indicate that the structure of guanine tetrads is possible. 

In 2010, Barthelmebs *et al.* [53] selected two aptamers for OTA, H8, and H12, each comprising 30 nucleotides. Fourteen rounds of SELEX were performed; each round consisted of incubating an ssDNA library, containing 10^15^ different sequences, with OTA modified magnetic beads (MBs). After washing, the beads were resuspended in water and the bound candidates were eluted from the target by heating for 1 min at 80 °C. ssDNA was PCR amplified, where a biotinylated antisense primer have been used to recover the sense strands through alkali denaturation on streptavidin coated MBs. The aptamers were selected in a binding buffer (PBS 1×, 1 mM MgCl_2_, 0.01% Tween 20, pH 7.4), where the dissociation constants were in a nanomolar range (96 nM for H12 and 130 nM for H8). A cross reactivity was tested using OTB and Phenylalanine as competitors for the binding to aptamers. Based on secondary structure characterization through m-fold program, the authors have demonstrated, by comparing the predicted structures of the selected aptamers and the aptamer 1.12.2, that the aptamers conserves two sequences. The first one, located in the stem region, was thought to be required in the stabilization of the structure due to the high GC percentage, while the second consensus sequence was located in the single-strand terminal loop. These results confirmed the importance of these two regions in the binding capacity of OTA. 

### 3.3. Aptamers *vs.* Antibodies

Antibodies have been widely used in immuno-based assays for OTA determination and have become indispensable in most tests required in food control [54,55]. Recently, the use of aptamers as biorecognition elements has improved the performances of biochemical methods. Although they mimic properties of antibodies, aptamers provide numerous advantages:

- Antibodies are identified through an *in vivo* process, requiring animal immunization, inducing an immune response against a target into a biological system. However, aptamer generation does not depend on *in vivo* conditions allowing the selection of aptamers against toxic or poor immunogenic targets.
- During antibody production, binding sites of the target are identified by the animal immune system. The identification is restricted by *in vivo* parameters. On the other side, SELEX conditions can be manipulated to select aptamers with the desired properties.
- The performance of antibodies can vary from batch to batch requiring optimizations with each new batch of antibodies. It is important to note that after selection, aptamers are chemically synthesized with extreme accuracy and reproducibility and purified under denaturating conditions to a very high degree of purity. Therefore, it is very rare to meet batch to batch variation problem.
- Due to their proteic nature, antibodies are very sensitive to temperature and pH variations and undergo irreversible denaturation. Unlike antibodies, aptamers are stable to high temperatures. They undergo reversible denaturation and could be renatured in few minutes. The function of immobilized aptamers is regenerable and they can be reused.
- During chemical synthesis, many modifications could be attached to aptamers for an easier immobilization on transducers enabling the design of several detection methods [56].

Thanks to their advantages, aptamers have been used as a good alternative to antibodies in many bioanalytical methods like ELISA (Enzyme Linked Immunosorbent Assay), affinity columns, and biosensors.

## 4. Molecular Interaction OTA-Aptamer

How aptamer recognizes OTA? Intramolecular base pairing defines higher-order structures. Therefore, a nucleic acid library of sequences is actually a library of three-dimensional shapes. Every candidate will display a unique combination of double-stranded helical segments, loops, and bulges. Each nucleotide may contribute in hydrogen bonds, electrostatic and van der Waals interactions. The scaffold, resulting from the intramolecular folding of oligonucleotides constituting the library, offers a three-dimensional potential for interacting with any type of target. No detailed structural characterization by Nuclear Magnetic Resonance (NMR) has been published yet, but several reports discussed the structure of aptamer after binding with OTA and the important bases involved in the recognition process. Cruz-agado and Penner used a strategy based on the displacement of an annealed oligonucleotide from an aptamer as a method to determine OTA concentration. In this work, the authors demonstrate that the bases (10 to 18) and (28 to 36) in the 5’ side play an important role during the recognition process [57]. A previous work by our group showed that the addition of OTA induced the formation of antiparallel G-quadruplex structure. The formation of antiparallel G-quadruplexes structure was directly related to the OTA concentration in the medium. This suggested that OTA possessed the ability of inducing the formation of antiparallel G-quadruplexes structure [58]. Castillo *et al.*, [59] draw the guanine quadruplex in OTA’s aptamer structure determined according to QGRS Mapper program. Later on, the Qiang Zhao group used a fluorescence method for detailed description of OTA-aptamer interaction. It has been found that the minimum number of nucleotides required in aptamer’s structure is 32 nucleotides, deleting 4 nucleotides at 3’end. The truncated aptamers with removal of one to three bases at 5’ end showed remarkably reduced binding affinity. These results confirmed the importance of the bases located in 5’ side in the specific recognition, while the four bases on the 3’end have a little effect on the affinity binding. The 24th and 23rd G bases on the aptamer sequence are also crucial for binding, as the substitution of G bases to C bases in these positions causes the loss of binding affinity of aptamer. Following, they introduced FAM (carboxyfluorescein) label at 3’, 5’end, or internal T bases (including T3, T8, T14, T19, and T30), using a single fluorophore-labeled aptamer for detection of OTA. This method relied on the change of the fluorescence intensity of the labeled dye induced by the specific binding of the fluorescent aptamer to OTA. The binding affinities of labeled internal T bases T3 and T14 (from 5’ end) were significantly reduced, as only small FA increase was observed at high concentrations of aptamer. These two T bases may play key role in maintaining correct conformation of the aptamer or in the interaction between aptamer and OTA.

Although, some papers already discussed the structure of aptamer after binding with OTA and the important bases during the recognition process, the detailed structural characterizations is still required.

## 5. Aptamer-Based Affinity Columns for Selective Retention of OTA

Because of the fluorescent properties of OTA, chromatographic techniques have been usually considered as reference methods for OTA analysis, mainly High Performance Liquid Chromatography with fluorescence detection (HPLC-FD). The chromatographic step is invariably preceded by sample purification which is usually based on the use of affinity columns. It consists of an affinity ligand immobilized on a gel packed into a plastic column. After conditioning, the sample is applied to the column and the target molecule is bound to the ligand by selective and reversible interactions. Finally, after washing the co-extractives, the analyte is eluted by breaking the bond ligand-target [60]. 

We focus in this review on aptamer-based affinity columns used for OTA purification (see Table 1). In addition to the advantages discussed above, the small size of aptamers and their ease immobilization on chromatographic supports, increase the density of immobilized aptamers on the support, thereby improving the density and the retention capacity. Indeed, stability is very important in extraction where strong elution conditions may be used [61]. 

### 5.1. Application to Cereals

Recently, after selecting a specific aptamer for OTA, Cruz-Agado and Penner have demonstrated the first aptamer affinity column (AAC) for concentration and separation of OTA [14]. The aptamer was conjugated to an agarose resin and packed into a column made with a pipet tip. The performance of the affinity column was first tested with a solution of OTA prepared in buffer, where the obtained extraction recovery was more than 97%. Afterwards, the feasibility of the method was confirmed with naturally contaminated wheat flour samples with a certified concentration of OTA. After fluorimetric determination, no significant difference was obtained between the concentrations determined with the AAC and the certified concentrations. In this study, the authors showed the high specificity of the developed AAC, where all the fluorescent interferences were removed during the washing steps. Indeed, the results were similar to that obtained with IACs with less dilution of the extraction solvent (four-fold instead of 10). In this work OTA concentrations were determined by fluorescence spectroscopy with a detection limit of 2 ng/g approximately. Despite the originality of the method, the reached detection limit is close to the maximum limits for OTA set by the European Commission. In another study, De Girolamo *et al.*, [62] have improved the procedure for preparation of AACs and proved their applicability to HPLC analysis of several wheat samples naturally contaminated with OTA. Average recoveries from wheat samples spiked at levels of 0.5–50 ng/g were found from 74% to 88% with detection and quantification limits of 0.023 and 0.077 ng/g, respectively. A comparative study was performed between several contaminated wheat samples using IAC and AAC showing a good correlation between the two procedures. The authors have also shown the ability to reuse the columns up to five times without affecting the binding affinity of the aptamer toward OTA. As a follow-up, the authors have combined the optimized AAC to a DNA-aptamer based-detection system for the direct (non-HPLC) determination of OTA in wheat. The detection was based on the measurement of time-resolved fluorescence response due to the interaction between terbium, a fluorescent lanthanide with a long lifetime, OTA, and the aptamer. The average recovery from samples spiked with 2.5–25 ng/g OTA was 77%, with a relative standard deviation lower than 6% and a quantification limit of 0.5 ng/g [63]. 

Another oligosorbent have been described by Wu *et al*. [64], where magnetic nanospheres (MNS) were used as solid support for aptamer immobilization. Nano-magnetic materials are very suitable for sample preparation, because they can be easily collected by a magnetic field with no need for centrifugation or filtration after extraction. After incubation of the sample with MNS, OTA formed a complex with the immobilized aptamer in a batch extraction procedure. Then, MNS were washed and OTA was eluted from the magnetic surface and quantified by HPLC-FD. The MNS-aptamer sorbent was used to clean up different samples of cereals products and wheat flour fortified with 2.5–50 ng/g where the recoveries varied from 67% to 90%. The authors have demonstrated, by comparing the chromatograms corresponding to the MNS-aptamer extraction with those of C18 SPE cartridge, that this method has the advantage of producing high purity extracts with less interference. Indeed this method reduced the use of organic solvents. 

### 5.2. Application to Beverages

Liquid matrices such as wines and beer are frequently diluted and filtered before being analyzed by HPLC or other detection methods. For that, it is of a great importance to apply aptamer-based affinity columns in OTA extraction, especially, from wine, which is considered as the second contributor to OTA intake. Chapius-Hugon *et al*., [65], have described an oligosorbent for OTA determination in red wine. The comparison of two immobilization procedures showed that the covalent binding of the aptamer using cyanogen-bromide-activated sepharose exhibited a higher binding efficiency and was more robust than the non-covalent binding using streptavidin-activated agarose. The oligosorbent was successfully applied for red wine sample spiked at 2 ng/mL of OTA. Indeed, it showed a high selectivity, in comparison to a conventional hydrophobic sorbent, and a good extraction recovery (93%). Another oligosorbent was reported by Rhouati *et al*. [66], using the covalent immobilization of OTA’s aptamer on cyanogen- bromide-activated sepharose, by decreasing the required concentration of aptamer to 0.2 mg/mL. The method was optimized for the extraction of OTA in spiked beer samples with an estimated detection limit of 0.2 ng/mL and an average recovery of 96%. By comparing the developed method to IACs, the authors showed that the oligosorbent can be reused several times with the same performance while the IACs had a low recovery yield after the second use. 

**Table 1:** Summary of the aptamer-affinity columns developed for Ochratoxin A (OTA) purification.

Sorbent	Matrix	Average recovery	Analysis	Reference
Agarose resin	Wheat	Non identified	Fluorescence spectroscopy	[14]
Agarose resin	Wheat	74% to 88%	HPLC-FD	[62]
Agarose resin	Wheat	77%	Time-resolved fluorescence	[63]
Magnetic nanospheres	Cereals and wheat flour	67% to 90%	HPLC-FD	[64]
Cyanogen-bromide activated sepharose	Wine	93%	HPLC-FD	[65]
Cyanogen-bromide activated sepharose	Beer	96%	HPLC-FD	[66]

The successful application of aptamers for the quantitative analysis of mycotoxins in food samples opens the way to significant improvements in quality control and food safety with regard to more dynamic testing of agricultural products.

## 6. Aptamers in OTA Biosensing

As alternative to immunochemical methods which suffer from several drawbacks, aptamer-based methods have been widely applied in OTA biosensing during recent years. As aptamers mimic the properties of antibodies, aptamer-based bioassays are analogous to immunoassays and can adopt different assay configurations to transduce bio-recognition events [67]. The reported aptamer-based methods for OTA detection vary from simple bioassays to sophisticated aptasensors. Herein, we summarize these methods taking into account the different transducers used (see Table 2).

### 6.1. Optical Methods

#### 6.1.1. Colorimetric

Several bioassays inspired by ELISA (Enzyme-Linked Immunosorbent Assay) technique, and using aptamers as biorecognition element have been reported. After selecting the aptamers H8 and H12, Barhtelmebs *et al*., [53] have developed the first Enzyme-Linked Aptamer Assay (ELAA), using the aptamers of OTA (H8, H12, and 1.12.2). Both strategies, direct and indirect competitive assays were investigated and compared to the traditional ELISA for OTA detection in wine samples. The direct assay was based on the competition between immobilized OTA and free OTA in the sample to bind to the labeled aptamer in the solution. In the indirect assay, free OTA competed with OTA-HRP conjugate for the immobilized aptamer. The direct competitive assay using the aptamer H12 showed the best midpoint value and detection limit. It was successfully applied in OTA detection in wine with a good sensitivity (1 ng/mL) and in less than 125 min. Furthermore, ELAA displayed similar characteristics with ELISA, but overcomes the limitations of antibodies which are unstable and expansive. Yang *et al.* [58] have described another aptamer-based assay using gold nanoparticules (AuNPs) as a colorimetric indicator. AuNPs are characterized by their high extinction coefficients and distance-dependant optical properties. The principle of this method is based on the fact that AuNPs are stable against salt-induced aggregation in the presence of OTA’s aptamer. However, the conformation of the aptamer changes after binding to OTA in a phosphate buffer containing Mg^2+^, leading to the deprotection and the aggregation of AuNPs which change the color from red to blue. The detection could be realized by monitoring the color change even with naked eye. This typical experiment needed only 5 min, however a preconcentration step is required to increase the sensitivity of the method, due to the high detection limit attained (8 ng/mL). Nevertheless, the same authors have reported a more sensitive colorimetric assay with a detection limit of 1 ng/mL. In this work, the biorecognition element was a nucleic acid hairpin structure, which includes G-rich oligonucleotides, OTA’s aptamer, and a blocking tail. G-rich oligonucleotides form G-quadruplexes, which bind specifically to hemin forming a horseradish peroxidase (HRP)-mimicking DNAzyme that may display a highly enhanced catalytic activity. The blocking tail captures a part of these sequences in the stem region of the hairpin inactivating the HRP-mimicking DNAzyme. After formation of the complex aptamer-OTA, the hairpin is opened and results in the self-assembly and activation of the DNAzyme. The developed method was applied on wine samples and showed a linear correlation with OTA concentration up to 4 ng/mL [68]. Very recently, the same group reported a new label-free colorimetric assay based on self-assembly of DNAzyme-aptamer conjugates. The structure of the new DNA includes the OTA-specific aptamer and a G-rich sequence of nucleotides mimicking peroxidase activity. The binding of OTA to aptamer results in the dehybridization between the oligonucleotides. Thus, the activity of the non-hybridized DNAzyme is linearly correlated with the concentration of OTA up to 12, with a limit of detection of 1.6 ng/mL [69].

Despite their efficiency, optical methods can suffer from several drawbacks, they require bulky and power-intensive light sources, detectors, and monochromators, and potential false signals arising from complex colored samples. Moreover, because the sensitivity of optical methods follows Lambert-Beer law, a minimum sample volume and path length is required to achieve certain performances [70].

#### 6.1.2. Fluorescent

Due to their high compatibility with nucleic acid aptamers, fluorescence techniques have been widely used in aptamer-based assays for OTA detection. Several quenchers and fluorophores exist, and can be easily attached to aptamers during or after the SELEX process. Indeed, the detection step can be carried out in real time without the need for the separation of target—probe complexes from unbound probes [71]. First, a fluorescent strip sensor based on aptamer-quantum dots technology has been reported for OTA analysis. QDs-labeled aptamers were migrated along a membrane strip to bind with two DNA probes on the test zone and control zone respectively. In the presence of OTA, the aptamer does not bind to DNA probe 1 in the test zone, while the aptamer would hybridize with the DNA probe 2 in the control zone. OTA was quantified by using the fluorescence intensity in both zones. The fluorescent sensor was applied for OTA detection in real red wine samples with a detection limit of 1.9 ng/mL [72]. Wang’s group has developed a fluorescent assay where OTA’s aptamer was labeled with FAM, while protected graphene was used as quencher. In the absence of OTA, the aptamer adsorbs onto the graphene by quenching FAM fluorescence. After OTA binding, the conformational change of the aptamer leads to the formation of G-quadruplex structures which are resistant to the adsorption. Therefore, the fluorescent intensity was proportional to OTA concentration in the sample. This method has been tested with 1% red wine containing buffer solution spiked with different concentrations of ochratoxin A [73]. Following the same principle, Guo *et al*. [74] have used single walled nanotubes as quencher, to construct a fluorescent aptasensor of OTA in beer. Compared to the sensor based on graphene, this approach eliminated the coating process and decreased unspecific interactions. However, the detection limit in both cases was higher than the regulatory standards of the European commission (7.48 and 9.64 ng/mL respectively). Duan *et al*. [75] have reported a fluorescent assay based on the conformational change of the aptamer upon OTA binding. The aptamer was immobilized on a microplate and its complementary oligonucleotide was labeled with FAM. The formation of the complex OTA-aptamer induces the dissociation of the complementary oligonucleotide, and thus decreases the fluorescence intensity. The method was successfully applied on maize flour samples with a detection limit of 0.01 ng/mL. Another fluorescent assay based on structure-switching signaling aptamer, was described by Chen *et al*. achieved [76]. This approach exploited the OTA-induced conformational change of aptamer, which resulted in the release of the hybridized quencher-tagged DNA strand from the fluorescein-labeled OTA aptamer, increasing fluorescence intensity. By applying the method in corn samples, a detection limit of 0.8 ng/mL was. Based on the fact that single stranded DNA oligonucleotides enhance Tb^3+^ emission and duplexes do not, Zhang *et al*. [77] have developed a signal-on fluorescent aptasensor for OTA detection in wheat samples. In this work, two DNA probes were added and hybridized with OTA’s aptamer. After OTA introduction, the probes were released enhancing the emission of Tb^3+^ and increasing the fluorescence intensity. The reported method allowed the detection of OTA with high sensitivity (LOD = 0.02 ng/mL). Recently, the direct detection of OTA using single fluorophore-labeled aptamer has been explored for the first time. This method relied on the change of the fluorescence intensity of the labeled dye induced by OTA binding. The authors demonstrated the feasibility of the method in red wine samples with a detection limit of 0.2 ng/mL [78]. Finally, Ma *et al*. [79] reported, for the first time, a femtogram ultrasensitive aptasensor for OTA detection by using fluorescence-based real-time quantitative PCR. A complementary DNA fragment to the aptamer was used as the template for the PCR amplification. The aptasensor was applied for OTA detection in red wine samples with good analyte concentration recovery. 

**Table 2:** Summary of the optical aptamer-based assays developed for OTA analysis.

Detection method	Working range (ng/mL)	LOD (ng/mL)	Sample	Sample preparation	Reference
**Colorimetric**	1–10	1	Wine	PVP*	[53]
	8–250	8	Wine	-	[58]
	1–4	1	Wine	Toluene, chloroform, Liquid-liquid extraction	[68]
	0.48–16	1.6	Wine	Toluene, chloroform, Liquid-liquid extraction	[69]
**Fluorescent**	1.9–10	1.9	Wine	-	[72]
	20–200	8.72	Wine	1% direct	[73]
	10–80	9.64	Beer	1% direct	[74]
	0.02–10000	0.01	Maize flour	Chloroform extraction	[75]
	1–100	0.8	Corn	Methanol:water extraction	[76]
	0.1–1	0.02	Wheat	Methanol:water extraction	[77]
	0.2–20	0.2	Wine	1% direct	[78]
**Luminescent**	0.02–3	0.007	Wheat	Methanol:water extraction, IAC	[80]
	0.0001–1	0.0001	Maize	Methanol:water extraction	[81]
	0.001–50	0.0003	Wheat	Methanol:water extraction	[82]

Note: *PVP: Poly vinyl pyrrolidone.

#### 6.1.3. Luminescent

Wang *et al*. [80] have developed the first electrochemiluminescent aptasensor for OTA determination. Electrochemiluminescence (ECL), combination of luminescence and electrochemical techniques, is one of the most sensitive methods used in biosensing. OTA’s aptamer was labeled with a signal producing compound (*N*-(4-aminobutyl)-*N*-ethyl-isoluminol) (ABEI) and hybridized to a complementary sequence immobilized on gold electrode. The introduction of OTA induces the dissociation of the labeled aptamer and thus the decrease of the ECL signal. The proposed method was applied to measure OTA in wheat samples with high sensitivity (0.007 ng/mL). Wu *et al*. [81] have used upconversion nanoparticules (UCNPs) as highly sensitive labels in the design of a luminescent bioassay for OTA detection in maize samples. The aptamer was immobilized on magnetic nanoparticules and hybridized to a complementary probe modified with UCNPs. This method was based on the decrease of the luminescent signal upon recognition of OTA by the aptamer. It allowed the determination of OTA with a low detection limit (0.0001 ng/mL). Based on the combination of target-induced strand release and cleavage of nicking endonuclease technology, an electrochemiluminescent bioassay for OTA detection was recently developed. The method was successfully applied in wheat samples where the detection limit attained 0.0003 ng/mL [82].

### 6.2. Electrochemical

Electrochemical methods present a promising alternative to the optical ones due to several advantages, such as, high specificity and sensitivity, small volume of samples required, possibility of miniaturization and automatization, low cost and less matrix interferences [70]. Therefore, electrochemical transduction has been widely used in OTA aptasensing, where we distinguish labeled and label-free detection methods (see Table 3). These methods were mostly based on amperometry (voltammetry) and Electrochemical Impedance Spectroscopy (EIS). The principle of amperometry is based on the measurement of a current, between the Working and Counter Electrode which is induced by a redox reaction at the Working electrode, under a fixed or variable (voltammetry) potential. In case of EIS, we follow the changes in electron-transfer resistance, over a range of frequencies, at the working electrode resulting from complexation [83].

#### 6.2.1. Labeled Aptasensors

In enzyme-labeled formats, the enzymatic reaction leads to the formation of an electroactive product generating an electrochemical signal which can be recorded by applying different electrochemical detections. First, Bonel *et al*. [84] have reported a competitive aptasensor for OTA detection in spiked wheat samples. OTA’s aptamer was immobilized on magnetic beads, and then HRP-labeled OTA and free OTA compete to bind to the immobilized aptamer. Modified MBs were placed onto a screen printed electrode (SPCE) under a magnetic field. After enzymatic reaction with the substrate, OTA determination was performed by Differential Pulse Voltammetry (DPV). The magnetic aptasensor showed a low limit of detection (0.07 ± 0.01 ng/mL). In this work, it has been shown that magnetic separations avoid unspecific adsorptions on electrode surface and allow high voltammetric currents resulting from the specific location of the beads on the working electrode. This strategy was also investigated by Barthelmebs *et al.*, where direct and indirect competitive aptasensors were reported. Two enzymes were used as labels, HRP and Alkaline Phosphatase (ALP). The aptasensor was successfully applied for wines samples with a detection limit of 0.11 ng/mL [85]. These two aptasensors were developed in batch conditions which could result in a loss of magnetic beads and thus, a lack of reproducibility. Recently, we integrated for the first time magnetic beads in the development of an automated flow-based aptasensor. The system was designed by injecting the functionalized MBs onto the surface of SPCE integrated into a central flow cell. The device was connected with a flow injection system and on-line detection of OTA in beer samples was performed amperometrically with a detection limit of 0.05 ng/mL. The use of an automated flow system reduces time analysis and avoids the human error [86].

Inorganic labels like (methylene blue) MB have also been used in electrochemical aptasensors for OTA. By labeling the aptamer with MB, in the absence of target, the aptamer is in the unfold form and MB is attached to the electrode leading to electron transfer. Afterwards, the binding induces a conformational change of the aptamer and inhibits the transfer of electrons producing electrical signals. On the basis of this concept, Kuang *et al*. reported an ultrasensitive aptasensor based on a competing model. Three single-stranded DNA molecules have been immobilized on the electrode surface, the aptamer (DNA2) was immobilized by base pairing to a DNA linker (DNA1) and a gold nanoparticle (Au NP)-functionalized DNA 3 which was used to amplify the sensing signal and improve the sensitivity. MB was used as the electrochemical redox probe, which interacted with DNA to label it, making the sensing current dependent on the amount of DNA on the electrode surface. OTA competed with DNA1 and DNA3 to combine with the aptamer, releasing DNA 2 and DNA3, and hence, reducing the peak current. The sensor was applied for wine samples with a detection limit of 0.03 ng/mL [87]. However, this method required multiple modifications and nanomaterial-based signal amplification treatments. In order to simplify the procedure, the same authors developed a one step aptasensor using thiol and MB dual labeled single stranded DNA probe (OTA’s aptamer). The formation of the complex aptamer-OTA induced the folding of the aptamer on the electrode surface which was monitored electrically. By increasing OTA concentration, the electron transfer is hindered decreasing the peak current. This aptasensor showed a very high sensitivity where the detection limit attained 0.00095 ng/mL [88]. 

#### 6.2.2. Label-Free Aptasensors

In order to avoid the complexity involved in labeling biomolecules, several examples have appeared in the literature for the development of label-free electrochemical aptasensors for OTA detection. Prabhakar *et al.* have reported an impedimetric aptasensor where OTA’s aptamer was covalently immobilized on mixed PANI-SA NB films. The aptasensor allowed detection of OTA by EIS with a detection limit of 0.1 ng/mL in a short time analysis of 15 min. Indeed, the authors have confirmed that this aptasensor can be reused 13 times, approximately [89]. However, this sensor has not been validated in real food samples. In another study, a thiolated aptamer was chemisorbed on gold nanoparticules for the development of EIS based aptasensor. The authors have shown the importance of calcium ions for the sensitivity of the sensor to OTA. They analyzed the sensitivity of various aptamers configurations, including aptamer homodimers. The aptasensor was regenerable in 1 mM HCl and it was validated with spiked coffee, flour, and wine samples. The detection limit was comparable to that of the HPLC method (0.048 ng/mL) [90]. 

We note in some reports, the use of signal amplification labels such as gold nanoparticules, or enzymes that have been demonstrated to give interesting results in terms of sensitivity, analysis time and correlation with standard tests [70]. In this concept, Tong *et al.* have inserted a new exonuclease-catalyzed target recycling strategy to achieve amplified electrochemical aptasensing of OTA. The reported label-free aptasensor was based on the immobilization of a ferrocene-labeled DNA probe and the complementary aptamer on a gold electrode. The formation of OTA-aptamer complex leads to the dissociation of aptamer from DNA probe which is transformed into a hairpin structure. Then, OTA is released for analyte recycling due to the use of exnonuclease, and DPV signal is produced with enhanced signal amplification. The developed method was validated with wheat starch sample with a high sensitivity (0.001 ng/mL) [91]. In another report, the same group described a dual signal amplification format based on OTA-mediated DNA circularization and the combination of rolling circle amplification and double signal probes introduction. The aptasensing platform was used for OTA determination in red wines and the detection limit attained 0.0002 ng/mL [92]. Despite the high sensitivity, signal amplification strategies require complicated procedures and expansive materials. 

**Table 3:** Summary of the electrochemical aptamer-based assays developed for OTA analysis.

Method	Working range (ng/mL)	LOD (ng/mL)	Sample	Sample preparation	Electrochemical detection	Reference
**Labeled Enzyme**	0.78–8.74	0.07 ± 0.01	Wheat	Extraction acetonitrile/water (6:4) (v/v)	DPV	[84]
	0.11–15	0.11	Wine	PVP, adjusting to pH7.2 after filtration	DPV	[85]
	0.05–2.56	0.05	Beer	Sonication, adjusting to pH7.4 after filtration	Amperometry	[86]
**Methylene Blue**	0.1–20	0.03	Wine	Solid phase extraction column	CV	[87]
	0.001–1	0.00095	Wine	No pretreatment	SWV	[88]
**Label-free**	0.1–10	0.1	-----	------------------------------------	EIS	[89]
	0.04–40	0.048	Coffee, flour, wine	10% (w/v) of sample matrix was spiked with OTA	EIS	[90]
	0.005–10	0.001	Wheat	Extraction acetonitrile: water (60/40) (v/v)	CV	[91]
	0.0005–10	0.0002	Wine	Extraction acetonitrile/water (6:4) (v/v)	SWSV	[92]
	0.00125–0.5	0.00025	Beer	Sonication, adjusting to pH 7.4 after filtration	EIS	[93]
	0.00012–0.0055	0.00012	Beer	Sonication, adjusting to pH 7.4 after filtration	EIS	[94]
	0.12–8.5	0.1	Beer	Sonication, adjusting to pH 7.4 after filtration	DPV	[95]

Notes: DPV: Differential Pulse Voltammetry; CV: Cyclic Voltammetry; SWV: Square Wave Voltammetry; SWSV: Square Wave Stripping Voltammetry; EIS: Electrochemical Impedance Spectroscopy.

Recently, our research group investigated an original strategy of immobilization in the production of a highly sensitive label-free aptasensor for OTA detection in beer samples. The immobilization of the aptamer was based on the combination of “click chemistry” and binary film grafting. This new strategy provides a uniform, controlled, and efficient immobilization improving the sensitivity and reducing the non specific signal. The impedimetric aptasensor showed a high sensitivity with a detection limit of 0.00025 ng/mL. Indeed, the authors demonstrated that the aptasensor is reusable at least up to 10 times without significant loss of performance [93]. The same authors have subsequently described an impedimetric and an amperometric label-free aptasensors, based on the aptamer’s conformational change upon target analyte binding. OTA’s aptamer was covalently immobilized on SPCE via a bifunctional spacer, forming diblock macromolecules. The spacer forms a long tunnel while the aptamer acts as a gate of the tunnel. After OTA binding, the gates were closed decreasing the electrochemical signal. Both aptasensors were successfully applied for beer samples where the detection limit was 0.00012 ng/mL by using PEG (Polyethylene Glycol) spacer [94] and 0.1 ng/mL by using hexamethyldiamine [95].

## 7. Future Perspectives: Other Mycotoxins

OTA was the first mycotoxin targeted by aptamer-based assays. After the promising results obtained, few reports have described the use of aptamers for the detection of other mycotoxins. First, McKeague *et al*. [96] have selected an aptamer sequence for the nephrotoxic mycotoxin fumonisin B_1_ (FB_1_). The aptamer displayed a dissociation constant in the nanomolar range and showed potential for use in fumonisin biosensors and solid phase extraction columns. Despite this, the selected aptamer was used only once in the development of an aptasensor for FB_1_ detection [97]. Wu *et al*. have also reported an aptasensor for the simultaneous detection of OTA and FB_1_ [98]. In 2012, a Canadian company (NeoVentures Biotechnology Inc., Ontario, Canada) obtained a patent for an Aflatoxin B_1_ (AFB_1_) aptamer and has produced a commercial affinity column and detection kit using the aptamer. Recently, this aptamer was used for the first time in the development of a chemiluminescent assay for AFB_1_ detection in corn samples [99]. Another aptasensor was also developed for aflatoxin M_1_ detection [100]. Moreover, a zearalenone (ZEN)-binding aptamer was selected very recently. It was successfully applied in the specific binding of ZEN in beer samples [101]. Finally, despite the numerous contaminating food mycotoxins that have been identified, we note that specific aptamers for only four of them have been selected. This can be explained by the fact that mycotoxins are small molecules, which makes SELEX procedures more complicated. The numerous advantages of aptamer-based assays open the door for the selection of new aptamers, affinity columns and detection kits for other mycotoxins threatening animal and human health. 

## 8. Conclusion

In conclusion, a broad range of aptamer-based assays models used for OTA extraction and detection in food analysis are available. The reported methods have attained high selectivity and sensitivity with a wide range of detection. Moreover, they are very rapid, cost-effective, and easy to use without requiring trained personnel. The use of aptamers as bio-recognition elements has overcome the principal limitation of antibodies which lack of stability. Most of the aptamer affinity columns and aptasensors described for OTA determination can be applied for very complex matrices with possible reuse for several times. Despite these advantages, only one aptamer-based kit for detecting OTA was commercialized by NeoVentures Biotechnology. The assay designated “OTA-Sense System”, consists of two components: an aptamer-based cleanup column and a detection solution. Therefore, there is still a challenge to develop more kits for OTA analysis and for simultaneous detection of different mycotoxins. 

## References

[1] Meerdink G.L. (2002). Mycotoxins. Clin. Tech. Equine Pract..

[2] Fernández-Cruz M.L., Mansilla M.L., Tadeo J.L. (2010). Mycotoxins in fruits and their processed products: Analysis, occurrence and health implications. J. Adv. Res..

[3] Van der Merwe K.S., Steyn P.S., Fourie L., DeScott B., Theron J.J. (1965). Ochratoxin A, a toxic metabolite produced by aspergillus ochraceus. Nature.

[4] International Agency for Research on Cancer (IARC) (1993). Ochratoxin A. Some Naturally Occurring Substances: Food Items and Constituents, Heterocyclic Aromatic Amines and Mycotoxins.

[5] (1995). Monographs on the Evaluation of Carcinogenic Risks to Humans.

[6] Boudra H., Le Bars P., Le Bars J. (1995). Thermostability of ochratoxin a in wheat under two moisture conditions. Appl. Environ. Microbiol..

[7] Turner N.W., Subrahmanyam S., Piletsky S.A. (2009). Analytical methods for determination of mycotoxins: A review. Anal. Chim. Acta.

[8] Van Dorst B., Mehta J., Bekaert K., Rouah-Martin E., De Coen W., Dubruel P., Blust R., Robbens J. (2010). Recent advances in recognition elements of food and environmental biosensors: A review. Biosens. Bioelectron..

[9] Radi A.-E., Muñoz-Berbel X., Cortina-Puig M., Marty J.-L. (2009). An electrochemical immunosensor for ochratoxin a based on immobilization of antibodies on diazonium-functionalized gold electrode. Electrochim. Acta.

[10] Prieto-Simon B., Campas M., Marty J.L., Noguer T. (2008). Novel highly-performing immunosensor-based strategy for ochratoxin a detection in wine samples. Biosens. Bioelectron..

[11] Lates V., Yang C., Popescu I., Marty J.-L. (2012). Displacement immunoassay for the detection of ochratoxin a using ochratoxin b modified glass beads. Anal. Bioanal. Chem..

[12] Remiro R., Ibanez-Vea M., Gonzalez-Penas E., Lizarraga E. (2010). Validation of a liquid chromatography method for the simultaneous quantification of ochratoxin a and its analogues in red wines. J. Chromatogr. A.

[13] Zhou S., Lai E.C., Miller J.D. (2004). Analysis of wheat extracts for ochratoxinA by molecularly imprinted solid-phase extraction and pulsed elution. Anal. Bioanal. Chem..

[14] Cruz-Aguado J.A., Penner G. (2008). Determination of ochratoxin a with a DNA aptamer. J. Agric. Food Chem..

[15] Duarte S.C., Pena A., Lino C.M. (2010). A review on ochratoxin A occurrence and effects of processing of cereal and cereal derived food products. Food microbiol..

[16] Ozden S., Akdeniz A.S., Alpertunga B. (2012). Occurrence of ochratoxin A in cereal-derived food products commonly consumed in turkey. Food Control.

[17] Araguás C., González-Peñas E., López de Cerain A. (2005). Study on ochratoxin a in cereal-derived products from spain. Food Chem..

[18] Tozlovanu M., Pfohl-Leszkowicz A. (2010). Ochratoxin a in roasted coffee from french supermarkets and transfer in coffee beverages: Comparison of analysis methods. Toxins.

[19] Cabañes F.J., Accensi F., Bragulat M.R., Abarca M.L., Castellá G., Minguez S., Pons A. (2002). What is the source of ochratoxin a in wine?. Int. J. Food Microbiol..

[20] Mateo R., Medina Á., Mateo E.M., Mateo F., Jiménez M. (2007). An overview of ochratoxin A in beer and wine. Int. J. Food Microbiol..

[21] Battilani P., Magan N., Logrieco A. (2006). European research on ochratoxin a in grapes and wine. Int. J. Food Microbiol..

[22] Mantle P.G., Chow A.M. (2000). Ochratoxin formation in aspergillus ochraceus with particular reference to spoilage of coffee. Int. J. Food Microbiol..

[23] Copetti M.V., Iamanaka B.T., Nester M.A., Efraim P., Taniwaki M.H. (2013). Occurrence of ochratoxin A in cocoa by-products and determination of its reduction during chocolate manufacture. Food Chem..

[24] Bircan C. (2009). Incidence of ochratoxin A in dried fruits and co-occurrence with aflatoxins in dried figs. Food Chem. Toxicol..

[25] Ozbey F., Kabak B. (2012). Natural co-occurrence of aflatoxins and ochratoxin A in spices. Food Control.

[26] Duarte S.C., Lino C.M., Pena A. (2011). Ochratoxin A in feed of food-producing animals: An undesirable mycotoxin with health and performance effects. Vet. Microbiol..

[27] Märtlbauer E., Usleber E., Dietrich R., Schneider E. (2009). Ochratoxin A in human blood serum—Retrospective long-term data. Mycotox. Res..

[28] Biasucci G., Calabrese G., Di Giuseppe R., Carrara G., Colombo F., Mandelli B., Maj M., Bertuzzi T., Pietri A., Rossi F. (2011). The presence of ochratoxin A in cord serum and in human milk and its correspondence with maternal dietary habits. Eur. J. Nutr..

[29] Pfohl-Leszkowicz A., Manderville R.A. (2007). Ochratoxin A: An overview on toxicity and carcinogenicity in animals and humans. Mol. Nutr. Food Res..

[30] Castegnaro M., Canadas D., Vrabcheva T., Petkova-Bocharova T., Chernozemsky I.N., Pfohl-Leszkowicz A. (2006). Balkan endemic nephropathy: Role of ochratoxins A through biomarkers. Mol. Nutr. Food Res..

[31] Pfohl-Leszkowicz A., Tozlovanu M., Manderville R., Peraica M., Castegnaro M., Stefanovic V. (2007). New molecular and field evidences for the implication of mycotoxins but not aristolochic acid in human nephropathy and urinary tract tumor. Mol. Nutr. Food Res..

[32] Khan M.A., Asrani R.K., Iqbal A., Patil R.D., Rottinghaus G.E., Ledoux D.R. (2013). Fumonisin b1 and ochratoxin A nephrotoxicity in japanese quail: An ultrastructural assessment. Comp. Clin. Pathol..

[33] Reddy L., Bhoola K. (2010). Ochratoxins—Food contaminants: Impact on human health. Toxins.

[34] Mally A., Hard G.C., Dekant W. (2007). Ochratoxin A as a potential etiologic factor in endemic nephropathy: Lessons from toxicity studies in rats. Food Chem. Toxicol..

[35] Pfohl-Leszkowicz A., Petkova-Bocharova T., Chernozemsky I.N., Castegnaro M. (2002). Balkan endemic nephropathy and associated urinary tract tumours: A review on aetiological causes and the potential role of mycotoxins. Food Addit. Contam..

[36] Pfohl-Leszkowicz A., Manderville R.A. (2012). An update on direct genotoxicity as a molecular mechanism of ochratoxin a carcinogenicity. Chem. Res. Toxicol..

[37] Al-Anati L., Petzinger E. (2006). Immunotoxic activity of ochratoxin A. J. Vet. Pharmacol. Ther..

[38] Froquet R., Le Drean G., Parent-Massin D. (2003). Effect of ochratoxin A on human haematopoietic progenitors proliferation and differentiation: An *in vitro* study. Hum. Exp. Toxicol..

[39] O’Brien E., Prietz A., Dietrich D.R. (2005). Investigation of the teratogenic potential of ochratoxin A and B using the fetax system. Birth Defects Res. Part B Dev. Reprod. Toxicol..

[40] Schwartz G. (2002). Hypothesis: Does ochratoxin A cause testicular cancer?. Cancer Causes Control.

[41] Wangikar P.B., Dwivedi P., Sinha N. (2004). Effect in rats of simultaneous prenatal exposure to ochratoxin A and aflatoxin B1. I. Maternal toxicity and fetal malformations. Birth Defects Res. Part B Dev. Reprod. Toxicol..

[42] Patil R.D., Dwivedi P., Sharma A.K. (2006). Critical period and minimum single oral dose of ochratoxin A for inducing developmental toxicity in pregnant wistar rats. Reprod. Toxicol..

[43] Jennings-Gee J.E., Tozlovanu M., Manderville R., Miller M.S., Pfohl-Leszkowicz A., Schwartz G.G. (2010). Ochratoxin A: In utero exposure in mice induces adducts in testicular DNA. Toxins.

[44] Commission of the European Communities (2012). Commission Recommendation (EC) No 594/2012 of 5 July 2012 emending regulation 1881/2006 as regards the maximum levels of the contaminants Ochratoxin A, non dioxin-like pcbs and melamine in foodstuffs. Off. J. Eur. Commun..

[45] Commission of the European Communities (2006). Commission Recommendation (EC) No 576/2006 of 17 August 2006 on the presence of deoxynivalenol, zearalenone, ochratoxin A, T-2 and HT-2 and fumonisins in products intended for animal feeding. Off. J. Eur. Commun..

[46] Kuiper-Goodman T., Hilts C., Billiard S.M., Kiparissis Y., Richard I.D.K., Hayward S. (2009). Health risk assessment of ochratoxin A for all age-sex strata in a market economy. Food Addit. Contam. Part A.

[47] Ellington A.D., Szostak J.W. (1990). *In vitro* selection of RNA molecules that bind specific ligands. Nature.

[48] Hermann T., Patel D.J. (2000). Adaptive recognition by nucleic acid aptamers. Science.

[49] Tombelli S., Minunni M., Mascini M. (2005). Analytical applications of aptamers. Biosens. Bioelectron..

[50] Toulmé J.-J., Da Rocha S., Dausse E., Azéma L., Lebars I., Moreau S. (2007). Les aptamères: Du concept à l’outil. Méd. Nucl..

[51] Cho M., Xiao Y., Nie J., Stewart R., Csordas A.T., Oh S.S., Thomson J.A., Soh H.T. (2010). Quantitative selection of DNA aptamers through microfluidic selection and high-throughput sequencing. Proc. Natl. Acad. Sci..

[52] Stoltenburg R., Reinemann C., Strehlitz B. (2007). Selex—A (r)evolutionary method to generate high-affinity nucleic acid ligands. Biomol. Eng..

[53] Barthelmebs L., Jonca J., Hayat A., Prieto-Simon B., Marty J.-L. (2011). Enzyme-linked aptamer assays (elaas), based on a competition format for a rapid and sensitive detection of ochratoxin A in wine. Food Control.

[54] Moises S.S., Schäferling M. (2009). Toxin immunosensors and sensor arrays for food quality control. Bioanal. Rev..

[55] Meulenberg E.P. (2012). Immunochemical methods for ochratoxin A detection: A review. Toxins.

[56] Jayasena S.D. (1999). Aptamers: An emerging class of molecules that rival antibodies in diagnostics. Clin. Chem..

[57] Cruz-Aguado J.A., Penner G. (2008). Fluorescence polarization based displacement assay for the determination of small molecules with aptamers. Anal. Chem..

[58] Yang C., Wang Y., Marty J.L., Yang X.R. (2011). Aptamer-based colorimetric biosensing of ochratoxin A using unmodified gold nanoparticles indicator. Biosens. Bioelectron..

[59] Geng X., Zhang D., Wang H., Zhao Q. (2013). Screening interaction between ochratoxin A and aptamers by fluorescence anisotropy approach. Anal. Bioanal. Chem..

[60] Senyuva H.Z., Gilbert J. (2010). Immunoaffinity column clean-up techniques in food analysis: A review. J. Chromatogr. B Anal Technol. Biomed. Life Sci..

[61] Zhao Q., Wu M., Le X.C., Li X.-F. (2012). Applications of aptamer affinity chromatography. TrAC Trends Anal. Chem..

[62] De Girolamo A., McKeague M., Miller J.D., DeRosa M.C., Visconti A. (2011). Determination of ochratoxin A in wheat after clean-up through a DNA aptamer-based solid phase extraction column. Food Chem..

[63] De Girolamo A., Le L., Penner G., Schena R., Visconti A. (2012). Analytical performances of a DNA-ligand system using time-resolved fluorescence for the determination of ochratoxin A in wheat. Anal. Bioanal. Chem..

[64] Wu X., Hu J., Zhu B., Lu L., Huang X., Pang D. (2011). Aptamer-targeted magnetic nanospheres as a solid-phase extraction sorbent for determination of ochratoxin A in food samples. J. Chromatogr. A.

[65] Chapuis-Hugon F., Du Boisbaudry A., Madru B., Pichon V. (2011). New extraction sorbent based on aptamers for the determination of ochratoxin A in red wine. Anal. Bioanal. Chem..

[66] Rhouati A., Paniel N., Meraihi Z., Marty J.-L. (2011). Development of an oligosorbent for detection of ochratoxin A. Food Control.

[67] Song S., Wang L., Li J., Fan C., Zhao J. (2008). Aptamer-based biosensors. TrAC Trends Anal. Chem..

[68] Yang C., Lates V., Prieto-Simon B., Marty J.L., Yang X. (2012). Aptamer-dnazyme hairpins for biosensing of ochratoxin A. Biosens. Bioelectron..

[69] Yang C., Lates V., Prieto-Simón B., Marty J.-L., Yang X. (2013). Rapid high-throughput analysis of ochratoxin A by the self-assembly of dnazyme-aptamer conjugates in wine. Talanta.

[70] Ricci F., Adornetto G., Palleschi G. (2012). A review of experimental aspects of electrochemical immunosensors. Electrochim. Acta.

[71] Nutiu R., Li Y. (2005). Aptamers with fluorescence-signaling properties. Methods.

[72] Wang L., Chen W., Ma W., Liu L., Ma W., Zhao Y., Zhu Y., Xu L., Kuang H., Xu C. (2011). Fluorescent strip sensor for rapid determination of toxins. Chem. Commun..

[73] Sheng L., Ren J., Miao Y., Wang J., Wang E. (2011). Pvp-coated graphene oxide for selective determination of ochratoxin A via quenching fluorescence of free aptamer. Biosens bioelectron..

[74] Guo Z., Ren J., Wang J., Wang E. (2011). Single-walled carbon nanotubes based quenching of free fam-aptamer for selective determination of ochratoxin A. Talanta.

[75] Duan N., Wu S.-J., Wang Z.-P. (2011). An aptamer-based fluorescence assay for ochratoxin A. Chin. J. Anal. Chem..

[76] Chen J., Fang Z., Liu J., Zeng L. (2012). A simple and rapid biosensor for ochratoxin A based on a structure-switching signaling aptamer. Food Control.

[77] Zhang J., Zhang X., Yang G., Chen J., Wang S. (2013). A signal-on fluorescent aptasensor based on Tb^3+^ and structure-switching aptamer for label-free detection of ochratoxin A in wheat. Biosens. Bioelectron..

[78] Zhao Q., Geng X., Wang H. (2013). Fluorescent sensing ochratoxin A with single fluorophore-labeled aptamer. Anal. Bioanal. Chem..

[79] Ma W., Yin H., Xu L., Xu Z., Kuang H., Wang L., Xu C. (2013). Femtogram ultrasensitive aptasensor for the detection of ochratoxin A. Biosens. Bioelectron..

[80] Wang Z., Duan N., Hun X., Wu S. (2010). Electrochemiluminescent aptamer biosensor for the determination of ochratoxin A at a gold-nanoparticles-modified gold electrode using *N*-(aminobutyl)-*N*-ethylisoluminol as a luminescent label. Anal. Bioanal. Chem..

[81] Wu S., Duan N., Wang Z., Wang H. (2011). Aptamer-functionalized magnetic nanoparticle-based bioassay for the detection of ochratoxin A using upconversion nanoparticles as labels. Analyst.

[82] Hun X., Liu F., Mei Z., Ma L., Wang Z., Luo X. (2013). Signal amplified strategy based on target-induced strand release coupling cleavage of nicking endonuclease for the ultrasensitive detection of ochratoxin A. Biosens. Bioelectron..

[83] Vidal J.C., Bonel L., Ezquerra A., Hernandez S., Bertolin J.R., Cubel C., Castillo J.R. (2013). Electrochemical affinity biosensors for detection of mycotoxins: A review. Biosens. Bioelectron..

[84] Bonel L., Vidal J.C., Duato P., Castillo J.R. (2011). An electrochemical competitive biosensor for ochratoxin A based on a DNA biotinylated aptamer. Biosens. Bioelectron..

[85] Barthelmebs L., Hayat A., Limiadi A.W., Marty J.-L., Noguer T. (2011). Electrochemical DNA aptamer-based biosensor for ota detection, using superparamagnetic nanoparticles. Sens. Actuators B Chem..

[86] Rhouati A., Hayat A., Hernandez D.B., Meraihi Z., Munoz R., Marty J.-L. (2013). Development of an automated flow-based electrochemical aptasensor for on-line detection of ochratoxin A. Sens. Actuators B Chem..

[87] Kuang H., Chen W., Xu D., Xu L., Zhu Y., Liu L., Chu H., Peng C., Xu C., Zhu S. (2010). Fabricated aptamer-based electrochemical “signal-off” sensor of ochratoxin A. Biosens. Bioelectron..

[88] Wu J., Chu H., Mei Z., Deng Y., Xue F., Zheng L., Chen W. (2012). Ultrasensitive one-step rapid detection of ochratoxin A by the folding-based electrochemical aptasensor. Anal. Chim. Acta.

[89] Prabhakar N., Matharu Z., Malhotra B.D. (2011). Polyaniline langmuir-blodgett film based aptasensor for ochratoxin A detection. Biosens. Bioelectron..

[90] Castillo G., Lamberti I., Mosiello L., Hianik T. (2012). Impedimetric DNA aptasensor for sensitive detection of ochratoxin A in food. Electroanalysis.

[91] Tong P., Zhang L., Xu J.J., Chen H.Y. (2011). Simply amplified electrochemical aptasensor of ochratoxin A based on exonuclease-catalyzed target recycling. Biosens. Bioelectron..

[92] Tong P., Zhao W.W., Zhang L., Xu J.J., Chen H.Y. (2012). Double-probe signal enhancing strategy for toxin aptasensing based on rolling circle amplification. Biosens. Bioelectron..

[93] Hayat A., Sassolas A., Marty J.-L., Radi A.-E. (2013). Highly sensitive ochratoxin A impedimetric aptasensor based on the immobilization of azido-aptamer onto electrografted binary film via click chemistry. Talanta.

[94] Hayat A., Andreescu S., Marty J.L. (2013). Design of peg-aptamer two piece macromolecules as convenient and integrated sensing platform: Application to the label free detection of small size molecules. Biosens. Bioelectron..

[95] Hayat A., Haider W., Rolland M., Marty J.L. (2013). Electrochemical grafting of long spacer arms of hexamethyldiamine on a screen printed carbon electrode surface: Application in target induced ochratoxin A electrochemical aptasensor. Analyst.

[96] McKeague M., Bradley C.R., De Girolamo A., Visconti A., Miller J.D., Derosa M.C. (2010). Screening and initial binding assessment of fumonisin B(1) aptamers. Int. J. Mol. Sci..

[97] Wu S., Duan N., Li X., Tan G., Ma X., Xia Y., Wang Z., Wang H. (2013). Homogenous detection of fumonisin B1 with a molecular beacon based on fluorescence resonance energy transfer between nayf4: Yb, ho upconversion nanoparticles and gold nanoparticles. Talanta.

[98] Wu S., Duan N., Ma X., Xia Y., Wang H., Wang Z., Zhang Q. (2012). Multiplexed fluorescence resonance energy transfer aptasensor between upconversion nanoparticles and graphene oxide for the simultaneous determination of mycotoxins. Anal. Chem..

[99] Shim W.-B., Mun H., Joung H.-A., Ofori J.A., Chung D.-H., Kim M.-G. (2014). Chemiluminescence competitive aptamer assay for the detection of aflatoxin B1 in corn samples. Food Control.

[100] Nguyen B.H., Tran L.D., Do Q.P., Nguyen H.L., Tran N.H., Nguyen P.X. (2013). Label-free detection of aflatoxin M1 with electrochemical Fe_3_O_4_/polyaniline-based aptasensor. Mat. Sci. Eng. C Mat. Biol. Appl..

[101] Chen X., Huang Y., Duan N., Wu S., Ma X., Xia Y., Zhu C., Jiang Y., Wang Z. (2013). Selection and identification of ssDNA aptamers recognizing zearalenone. Anal. Bioanal. Chem..

